# Multi-scale encoding of amino acid sequences for predicting protein interactions using gradient boosting decision tree

**DOI:** 10.1371/journal.pone.0181426

**Published:** 2017-08-08

**Authors:** Chang Zhou, Hua Yu, Yijie Ding, Fei Guo, Xiu-Jun Gong

**Affiliations:** 1 School of Computer Science and Technology, Tianjin University, Tianjin, China, 300072; 2 Tianjin Key Laboratory of Cognitive Computing and Application, Tianjin, China, 300072; Harbin Institute of Technology Shenzhen Graduate School, CHINA

## Abstract

Nowadays a number of computational approaches have been developed to effectively and accurately predict protein interactions. However, most of these methods typically perform worse when other biological data sources (e.g., protein structure information, protein domains, or gene neighborhoods information) are not available. In the present work, we propose a method for predicting protein interactions making full use of physicochemical characteristics of amino acids. A protein sequence is encoded at multi-scale by seven properties, including their qualitative and quantitative descriptions, of amino acids. Five kinds of protein descriptors, frequency, composition, transformation, distribution and auto covariance, are extracted from these encodings for representing each protein sequence. The new formed feature representation consisted of 347 dimensions is able to capture not only the compositional and positional information but also their statistical significance of amino acids in the sequence. Based on such a feature representation, the gradient boosting decision tree algorithm is introduced to predict protein interaction class. When the proposed method is tested with the PPI data of *S.cerevisiae*, it achieves a prediction accuracy of 95.28% at the Matthew’s correlation coefficient of 90.68%. Compared with the state-of-the-art works on *H.pylori* and *Human*, the accuracies can be raised to 89.27% and 98.00% respectively. Extensive experiments are performed for a crossover protein-protein interactions network and the prediction accuracies are also very promising. Because of learning capabilities of the gradient boosting decision tree and the mutil-scale feature representation scheme, the proposed method might be a useful tool for future proteomics studies.

## Introduction

Protein-protein interactions (PPIs) play a key role in various biological functions such as DNA transcription, metabolic cycles and signaling cascades in cells. Therefore, identification of PPIs can provide a great insight into protein functions and further biological processes [1]. With the development of proteomics, many experimental techniques have been developed such as protein chip [2], tandem affinity purification (TAP) [3] and other high-throughput biological techniques [4]. However, PPI pairs identified by experimental approaches only cover a small fraction of the whole PPI networks [5]. In addition, they hold inherent disadvantages, such as being time-consuming, expensive, and having high false positive rate. Hence, there is a strong motivation to develop efficient computational methods as alternative for inferring PPIs efficiently and accurately [6, 7].

A number of computational methods have been developed for the prediction of PPIs. However the application of most existing methods is limited because they need information about protein homology or the interaction marks of the protein partners. Recently, much effort has been devoted to propose machine learning approaches for detecting PPIs using protein sequences alone [8–10].

For predicting PPIs by sequences, one of the main computational challenges is to find a suitable way to fully describe the important information of PPI. Shen et. al [8] used the conjoint triad method to extract features of protein sequences based on properties of amino acids. They classified 20 amino acids into seven group according to dipoles and volumes of the side chains to reduce the dimensions of vector space. The traid types and their numerical values of three continuous amino acids are feed into the feature vector space. Zhou [10] and Yang [11] divided the whole sequences into different local regions of varying length, then calculated three local descriptors (composition, transition and distribution) in each local region to describe multiple overlapping continuous and discontinuous interaction patterns in protein sequences. Guo et. al [9] used the auto covariance (AC) method to construct the feature vectors of protein sequences. It took neighboring effects into account and discovered patterns in entire sequences. Furthermore, there are several other kinds of feature representation methods including Auto Cross Covariance (ACC) [9], Multi-scale Continuous and Discontinuous (MCD) [12], and Multi-scale Local Feature Representation (MLD) [13]. Fortunately enough, recent advances in developing numerous web servers for extracting features from biological sequences, such as RepDNA [14], RepRNA [15] and Pse-in-One [16] for DNA, RNA and protein sequence respectively, make the procedure quickly and effectively.

Sample classification is another important issue for predicting PPIs computationally. Most of current computational methods are based on the traditional classifier such as support vector machine [9, 10, 12] and random forests [13, 17]. Although these classifiers have strong classification ability, they need much labor and time to adjust corresponding parameters for the best performance. Recently, Gradient Boosting Decision Tree (GBDT) [18] classifier is earning reputation for its powerful classification performance. As an effective off-the-shelf method for generating models for classification and regression tasks, GBDT produces a prediction model in the form of an ensemble of weak prediction models, builds the model in a stage-wise fashion, and generalizes them by allowing optimization of an arbitrary differentiable loss function. Because of the arbitrary of choosing the loss function, it makes the GBDT highly customizable to any particular data-driven task. Meanwhile, the GB algorithms are relatively simple to implement, which allows one to experiment with different model designs. Thus the GBDT algorithms have shown considerable success in not only practical applications [19], but also in various machine learning and data mining challenges [20].

In this paper, we present a computational approach for predicting PPIs by combining a multi-scale encoding representation of proteins and a gradient boosting decision tree classifier. First, physicochemical characteristics, including their qualitative and quantitative attributes, of amino acids are used to encode a protein sequence at multi-scale. Then, Five kinds of protein descriptors, frequency, composition, transformation, distribution and auto covariance, are extracted from these encodings for representing each protein sequence. A 347 dimensional vector of a protein sample is obtained after the transformation. Thirdly, we combine every two corresponding protein feature vectors into 694-dimensional vectors as the inputs for classifier. Finally, the gradient boosting decision tree algorithm is introduced to predict protein interaction class based on the multi-scale feature representation scheme.

In order to evaluate the performance of the proposed method, it is tested with the PPI data of *S.cerevisiae*. The prediction accuracy of 95.28% and Matthew’s correlation coefficient of 90.68% are achieved. Compared with the state-of-the-art works on *H.pylori* and *Human*, the accuracies can be raised to 89.27% and 98.00% respectively. Extensive experiments are performed for a crossover protein-protein interaction network and the prediction accuracies are also very promising.

## Results

In this section, we firstly evaluate the performance of the proposed method for predicting PPIs on three different PPI datasets: *S.cerevisiae*, *H.pylori* and *Human* by using different evaluation measures including Matthew’s correlation coefficient (MCC). Then, the prediction performances on three different feature representations including qualitative characteristic feature, quantitative characteristic feature, and the full features are discussed. Thirdly, we compare the classification performances among GBDT, Random Forest(RF) and Support Vector Machine(SVM) by using the same feature vector representation. Furthermore, we compare the performance of the proposed method with the previous existing methods. In addition, we also present the results of the experiments on a crossover protein-protein interaction network.

### Data set

The PPI datasets from *S.cerevisiae*, *H.pylori* and *Human* are used to evaluate the performances.

All the datasets are downloaded from the existing works done by You et. al [12], Martin et. al [21] and Huang et. al [22] respectively. The distributions of the golden positive and negative samples (GPS and GNS) are shown in Table 1.

**Table 1:** The distributions of the goldend positive and negative samples.

Dataset	#GPS	#GNS	#Total
*S.cerevisiae*	5594	5594	11188
*H.pylori*	1458	1458	2916
*Human*	3899	4262	8161

It should be noticed that the sequence homology is an important problem for sequence-based predictors [23]. All the protein pairs which contain a protein with fewer than 50 residues or have ≥ 40% sequence identity have been removed in the first dataset. The third dataset has removed protein pairs with ≥ 25% sequence identity. In the second dataset, the positive samples were from proteome-wide experiment using two-hybrid measurements, the negative samples were selected randomly. For testing the generability of models, sequence redundancy in this dataset was not considered.

In the first two datasets, the numbers of positive and negative samples are equal. For the third dataset, the number of positive samples is less than the one of negative samples. We choose these three balanced and unbalanced datasets for testing the generability of our model.

### Evaluation measures

To evaluate the performance of the proposed method, five-fold cross validation and a couple of assessment measures are used in this study. These criteria includes overall prediction accuracy (ACC), sensitivity(SN), positive predictive value (PPV), weighted average of the PPV and F-score, and Matthew’s correlation coefficient (MCC). There are defined in Eqs from 1 to 5.

$$\begin{array}{c} \text{ACC}=\frac{TP+TN}{TP+FP+TN+FN} \end{array}$$ (1)

$$\begin{array}{c} \text{SN}=\frac{TP}{TP+FN} \end{array}$$ (2)

$$\begin{array}{c} \text{PPV}=\frac{TP}{TP+FP} \end{array}$$ (3)

$$\begin{array}{c} F_{\text{score}}=2\times \frac{SN\times PPV}{SN+PPV} \end{array}$$ (4)

$$\begin{array}{c} \text{MCC}=\frac{TP\times TN-FP\times FN}{\sqrt{\left(TP+FN)\times (TN+FP)\times (TP+FP)\times (TN+FN\right)}} \end{array}$$ (5)

where true positive (TP) is the number of true PPIs that are predicted correctly; false negative (FN) is the number of true PPIs that are predicted to be non-interacting pairs; false positive (FP) is the number of true non-interacting pairs that are predicted to be PPIs, and true negative (TN) is the number of true non-interacting pairs that are predicted correctly.

### Prediction performances of the proposed method

The performances of the proposed approach are investigated using the PPI datasets of three species: *S.cerevisiae*, *H.pylori* and *Human*. To make the experimental results generalizable regarding new data in the predictions, each dataset is randomly partitioned into training and testing sets via a five-fold cross validation. Each of the five subsets acts as an independent holdout testing dataset for the model trained with the rest of four subsets. Thus five models for each dataset are generated for its corresponding five sets of data.

The prediction performances of GBDT classifier with full feature representation of protein sequences across five runs is shown in Tables 2–4. The highest prediction accuracies on three PPI datasets are 95.84%, 91.94% and 98.41% respectively. The average ones on them reach 95.28%, 89.27% and 98.00%. These results show that the performance of the proposed method is quite promising. To better investigate the prediction ability of our model, we also calculated the values of SN, PPV, and MCC. Nearly over 90% of these values on three datasets ensures robustness of the prediction capability of the method.

**Table 2:** Five-fold cross-validation on *S.cerevisiae* dataset.

Testset	ACC%	SN%	PPV%	F-score%	MCC%
1	95.22	92.75	97.79	95.20	90.57
2	95.00	91.96	98.02	94.90	90.17
3	94.77	91.99	97.45	94.64	89.69
4	95.57	92.86	98.09	95.40	91.27
5	**95.84**	**94.21**	**97.16**	**95.66**	**91.71**
Average±Std	95.28±0.38	92.75±0.81	97.18±0.62	97.70±2.22	90.68±0.72

**Table 3:** Five-fold cross-validation on *H.pylori* dataset.

Testset	ACC%	SN%	PPV%	F-score%	MCC%
1	89.21	92.00	88.99	90.47	78.11
2	87.14	90.32	84.00	87.05	74.50
3	88.34	**93.31**	84.39	88.63	77.13
4	**91.94**	91.67	**91.99**	**91.83**	**83.87**
5	89.71	87.94	90.51	89.21	79.41
Average±Std	89.27±1.59	91.05±1.82	87.98±3.23	89.44±1.62	78.60±3.08

**Table 4:** Five-fold cross-validation on *Huamn* dataset.

Testset	ACC%	SN%	PPV%	F-score%	MCC%
1	98.16	97.05	99.08	98.05	96.33
2	97.18	95.35	98.83	97.06	94.41
3	**98.41**	**97.62**	**99.11**	**98.36**	**96.82**
4	98.35	97.47	98.92	98.19	96.67
5	97.92	97.04	98.56	97.79	95.83
Average±Std	98.00±0.44	96.90±0.81	98.90±0.19	97.89±0.45	96.01±0.87

To further investigate the performance on the different numbers of positive and negative samples, we analyze the standard variances of the prediction accuracies on three datasets. The second dataset gets the highest standard variance and lower value of MCC. This might be caused by the sequence redundancy problem.

### Prediction performances with different features

In order to understand the contribution of QLC and QNC features, the investigated experiments are performed on the three datasets using three kinds of feature components, QLC, QNC and QLC+QNC. Also, the five-fold cross validation is used to evaluate the performance. The results are shown in Tables 5–7.

**Table 5:** Contribution of QLC, QNC and QLC+QNC on *S.cerevisiae* dataset.

Feature	ACC%	SN%	PPV%	F-score%	MCC%
QLC	94.63±0.30	91.23±0.50	97.89±0.13	94.44±0.21	89.46±0.54
QNC	94.68±0.40	92.06±0.55	97.16±0.41	94.54±0.42	89.48±0.81
QLC+QNC	95.28±0.38	92.75±0.81	97.18±0.62	97.70±2.22	90.68±0.72

**Table 6:** Contribution of QLC, QNC and QLC+QNC on *H.pylori* dataset.

Feature	ACC%	SN%	PPV%	F-score%	MCC%
QLC	88.10±0.74	88.35±1.07	87.92±1.90	88.12±0.86	76.23±1.45
QNC	88.17±1.18	90.75±1.18	86.33±2.32	88.46±1.11	76.47±2.28
QLC+QNC	89.27±1.59	91.05±1.82	87.98±3.23	89.44±1.62	78.60±3.08

**Table 7:** Contribution of QLC, QNC and QLC+QNC on *Huamn* dataset.

Feature	ACC%	SN%	PPV%	F-score%	MCC%
QLC	97.74±0.45	96.38±0.85	98.84±0.21	97.59±0.50	95.48±0.90
QNC	97.87±0.13	96.43±0.40	99.08±0.18	97.74±0.16	95.75±0.25
QLC+QNC	98.00±0.44	96.90±0.81	98.90±0.19	97.89±0.45	96.01±0.87

On the *S.cerevisiae* dataset, QLC and QNC demonstrate similar performance and their combination outperforms other two in all the performance measures, especially F-score by 3.26% improvement. Moreover, the values of its MCC and SN can be raised at least by 1.22%, and 1.52%. The similar situations are true on the datasets of *H.pylori* and *Huamn*.

These comparative experiments show that QLC and QNC play a similar role, are somehow complementary, in the prediction of PPIs. And their combination improve significantly the performance.

### Comparsion of the prediction performance with different classifiers

**Fig 1:**
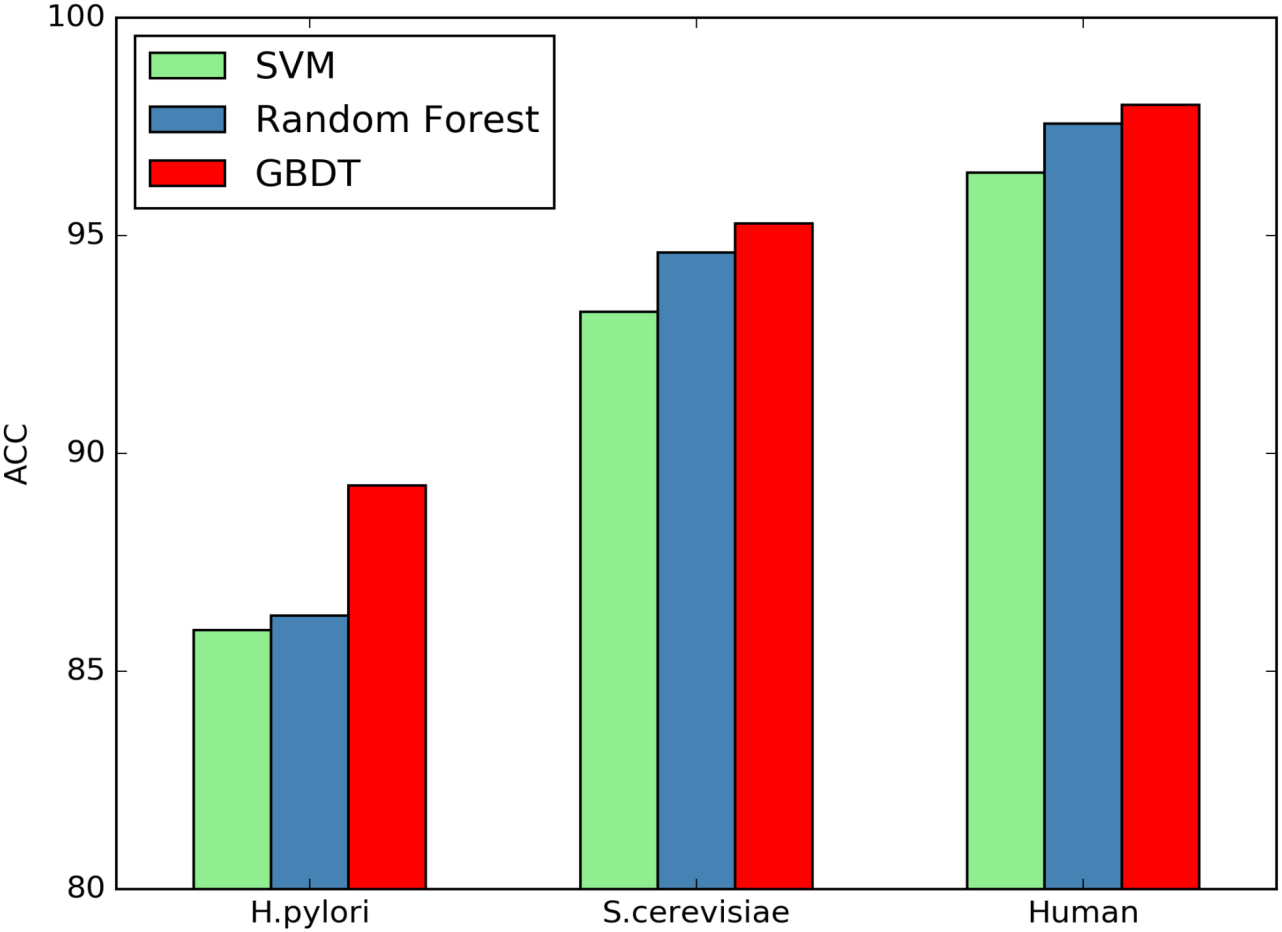
Comparison of ACC by different classifiers.

**Fig 2:**
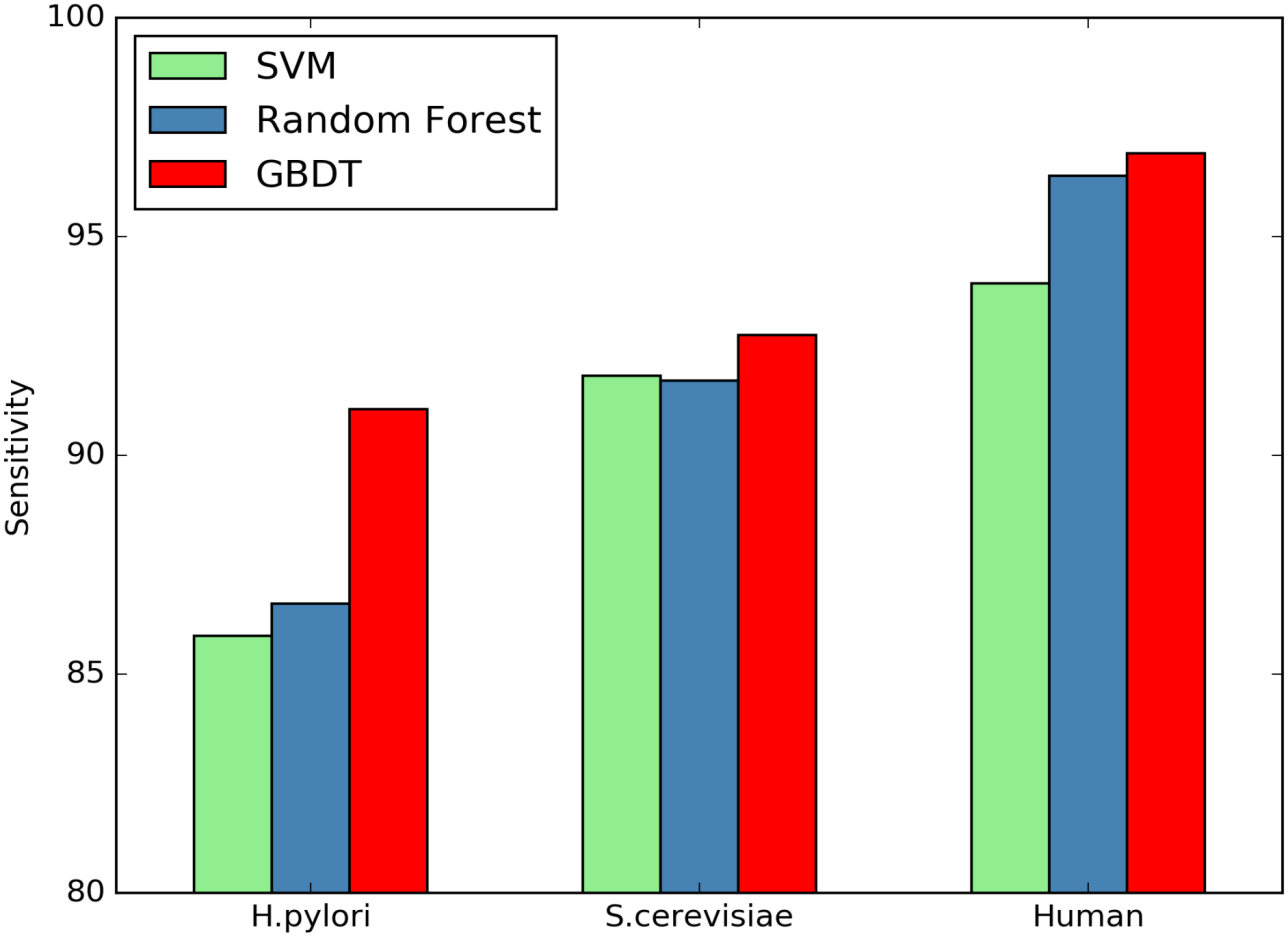
Comparison of SN by different classifiers.

**Fig 3:**
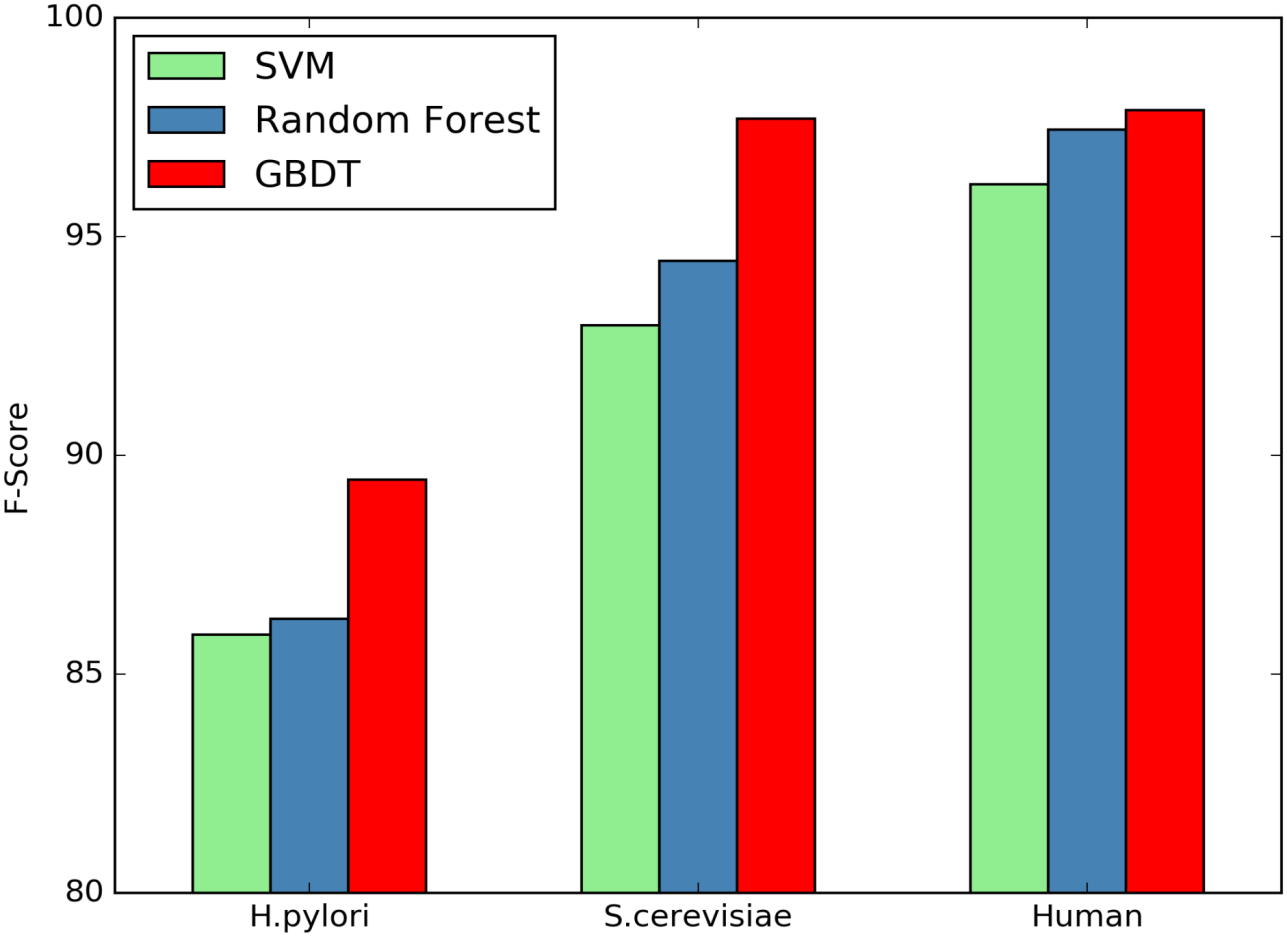
Comparison of F-Score by different classifiers.

**Fig 4:**
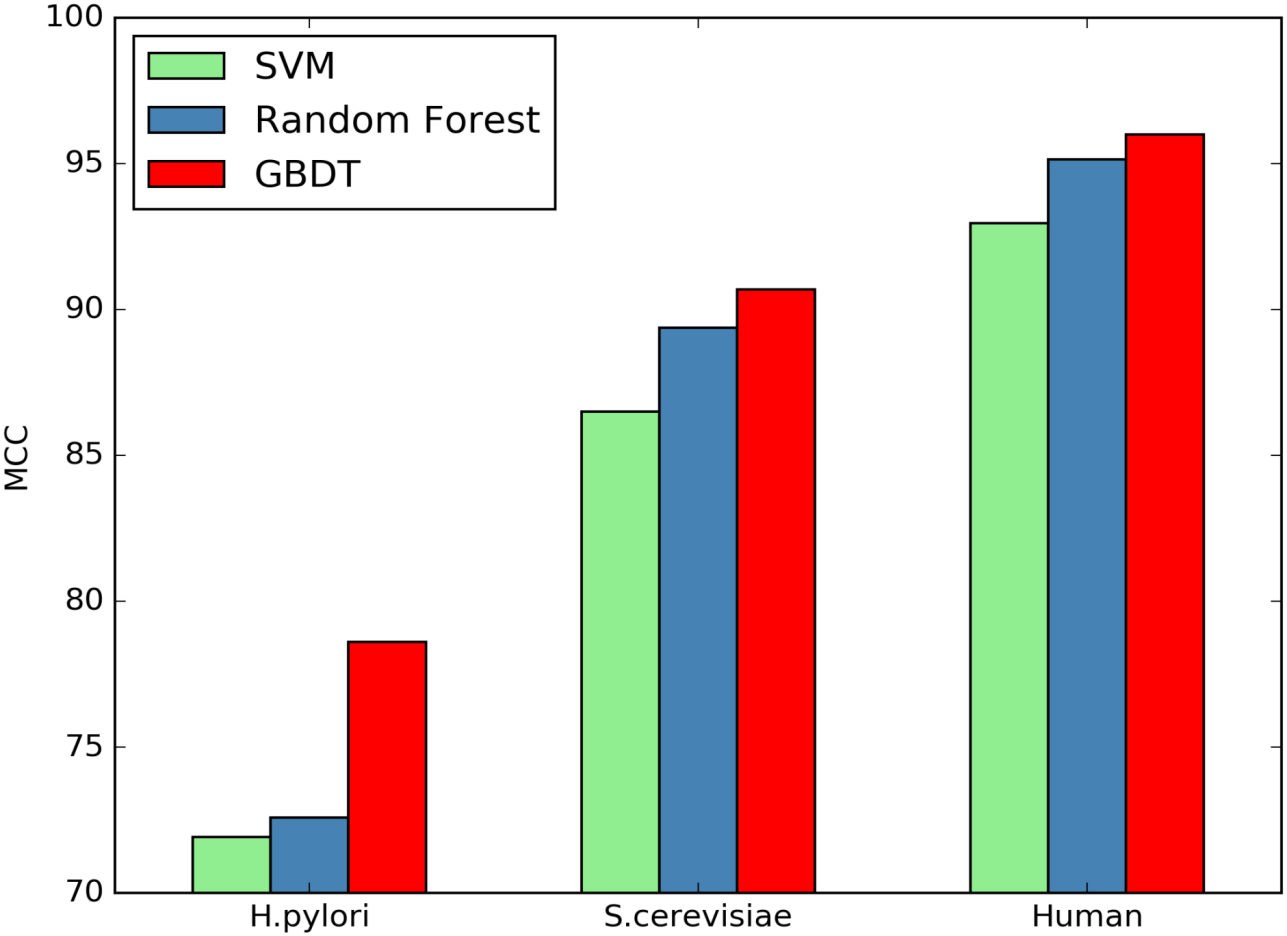
Comparison of MCC by different classifiers.

Here we investigate whether or not the GBDT classifiers can significantly improve the performance of PPI prediction compared against other classifiers. SVM and Random Forest are two commonly used classifiers for predicting protein interactions. We compare the classification performance between SVM, Random Forest and GBDT using the same features. Figs 1–4 plot accuracy, sensitivity, F-score and MCC value for the three classifiers.

As shown in Table 8, the GBDT wins all other two classifiers on the three datasets in terms of all the assessment measures. Compared with SVM On the *H.pylori* dataset, the prediction accuracy of GBDT is nearly 3.33% higher, the SN value is improved from 85.87% to 91.05%, and MCC value can be raised by 6.69%. Further, compared with Random Forest, the accuracy is improved from 86.28% to 89.27%, SN and MCC are increased by 4.44% and 6.02%, respectively. The similar situation occurs for other two datasets.

**Table 8:** Performance comparison using different classifiers on three datasets.

*Species*	Classifier	ACC%	SN%	PPV%	F-score%	MCC%
*S.cerevisiae*	SVM	93.25	91.82	94.16	92.97	86.51
	RF	94.61	91.71	97.34	94.44	89.37
	GBDT	95.28	92.75	97.18	97.70	90.68
*H.pylori*	SVM	85.94	85.87	86.00	85.90	71.91
	RF	86.28	86.61	86.01	86.27	72.58
	GBDT	89.27	91.05	87.98	89.44	78.60
*Huamn*	SVM	96.45	93.93	98.58	96.20	92.96
	RF	97.57	96.39	98.51	97.44	95.15
	GBDT	98.00	96.90	98.90	97.89	96.01

### Comparison of the prediction performance with existing methods

In order to highlight the advantage of our method, we compare the prediction ability with the state-of-the-art methods on the PPI data of *H. pylori* and *S.cerevisiae*. These methods include Ding et. al [17, 24], You et. al [12], Wong et. al [25], Guo et. al [9], and zhou et. al [10]. The features, feature extraction methods and classifiers used in these method are shown in Tables 9 and 10.

**Table 9:** The performance of different methods on *S.cerevisiae* dataset.

Method	Feature	Classifier	ACC%	SN%	PPV%	MCC%
Our	QLC+QNC	GBDT	**95.28**	92.28	97.90	**90.68**
Ding	HOG+SVD	RF	94.83	**92.40**	97.10	89.77
You	MLD	RF	94.72	94.34	**98.91**	85.99
You	AC+CT+LD+MAC	E-ELM	87.00	86.15	87.59	77.36
You	MCD	SVM	91.36	90.67	91.94	84.21
Wong	PR-LPQ	Rotation F	93.92	91.10	96.45	88.56
Gou	ACC	SVM	89.33	89.93	88.87	NA
Gou	AC	SVM	87.36	87.30	87.82	NA
Zhou	LD	SVM	88.56	87.37	89.50	77.15
Yang	LD	KNN	86.15	81.03	90.24	NA

**Table 10:** The performance of different methods on *H.pylori* dataset.

Method	ACC%	SN%	PPV%	MCC%
Our	**89.27**	91.05	87.98	78.62
Ding’s work(HOG+SVD)	89.06	88.15	**89.79**	78.15
Ding’s work(MMI+NMBAC)	87.59	86.81	88.23	75.24
You’s work(MLD)	88.30	**92.47**	85.99	**79.19**
You’s work(AC+CT+LD+MAC)	87.50	88.95	86.15	78.13
You’s work(MCD)	84.91	83.24	86.12	74.40
Huang’s work(DCT+SMR)	86.74	86.43	87.01	76.99
Zhou’s work	84.20	85.10	83.30	NA

On the *S.cerevisiae* dataset, the prediction accuracy of our model increases nearly by 1% than the best method with the highest MCC, and slight low values of SN and PPV. On the *H.pylori* dataset, our model also obtain the best prediction accuracy with nearly similar values of SN, PPV, and MCC as ones in the best methods. These experimental results demonstrate that our model outperforms all other previous methods on a couple of PPI datasets.

### Prediction performance on a real Wnt-related network

**Fig 5:**
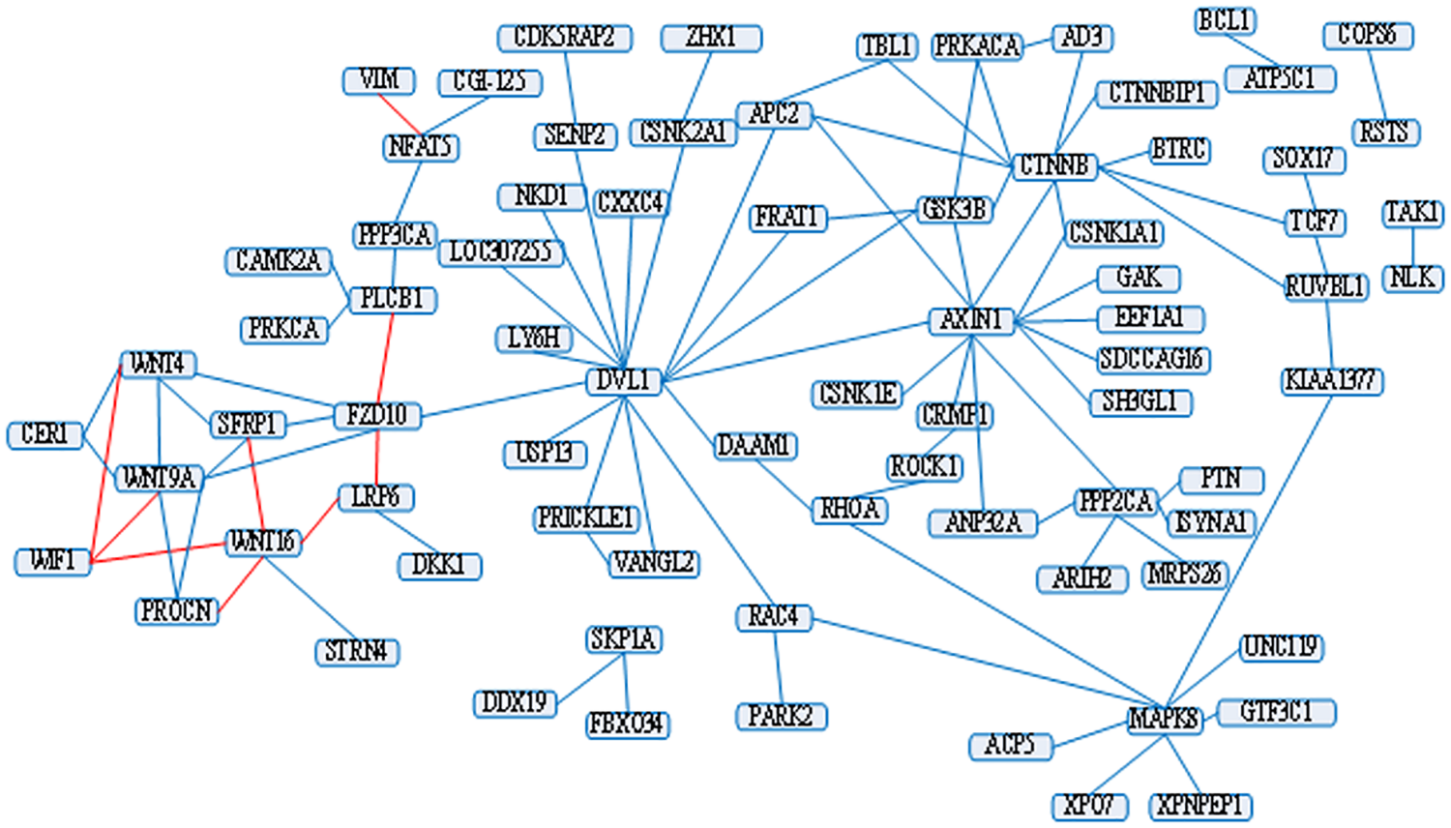
The prediction on the Wnt-related pathway network.

The most useful application for predicting PPIs is to build a biological meaningful PPI network. To test the generability of our method, the model trained on the *S.cerevisiae* dataset is applied to a real Wnt-related network produced by Ulrich et. al [26]. It predicts 87 interactions among all the 96 PPI pairs, see Fig 5 (the red line indicates a false prediction). Compared to 73 interactions by Shen’ method [8], the accuracy of our method raises by 14.58%.

Our result is also compared against Ding’s work [24] with 91 interactions in this network. We find that the false predictions between two methods are completely different. Meanwhile, our 9 false predictions connect 10 proteins and their 5 false predictions connect 9 proteins. These slight differences might suggest that the two methods could apply to different situations.

To further explore the false predictions, we find that three proteins (FZD10, WNT9A and WNT4) are often predicted incorrectly via different runs. FZD10 is a receptor for Wnt proteins, which may be involved in transduction and intercellular transmission of polarity information. WNT9A and WNT4 are ligands for the members of the frizzled family of seven transmembrane receptors, which are likely to signal over only few cell diameters. It is hypothesized that the poor signal interaction between proteins that transmit a small amount of signal at the cell diameter and other proteins will result in poor prediction performance.

## Discussions

It should be noticed that high dimension data might cause over-fitting, information redundancy and dimension disaster, which can overestimate the performance and reduce the generalization ability of a predictor [27]. To exclude noise or redundant information, Yang and Chen et al [28, 29] employed ANOVA, Zhao et al [30] used mRMR program to further optimize the feature set. A series of feature sets in various sizes were obtained based on IFS strategy. However, GBDT is an additive model that minimizes the loss function by the weak classifier. With this model, the individual classifiers do not need to be particularly complex. On the contrary, simple classifiers tend to work best to evade from overfitting. Furthermore, the optimal value of iterations and the total number of leaves are often selected by monitoring prediction error. Moreover GBDT selects features in the form of an ensemble of decision trees.

As shown in a series of recent publications, in addition to the predictor’s high accuracy, it is also very important to make its web-server available so that users can easily get the results without the need to go through the mathematical details [31–35]. Only with this, can it be widely used by most experimental scientists [36]. All the source codes are available at the github server (https://github.com/lovekeyczw/zhouchang/). we shall make efforts in our future work to provide a web-server for the method reported in this paper.

## Methods

**Fig 6:**
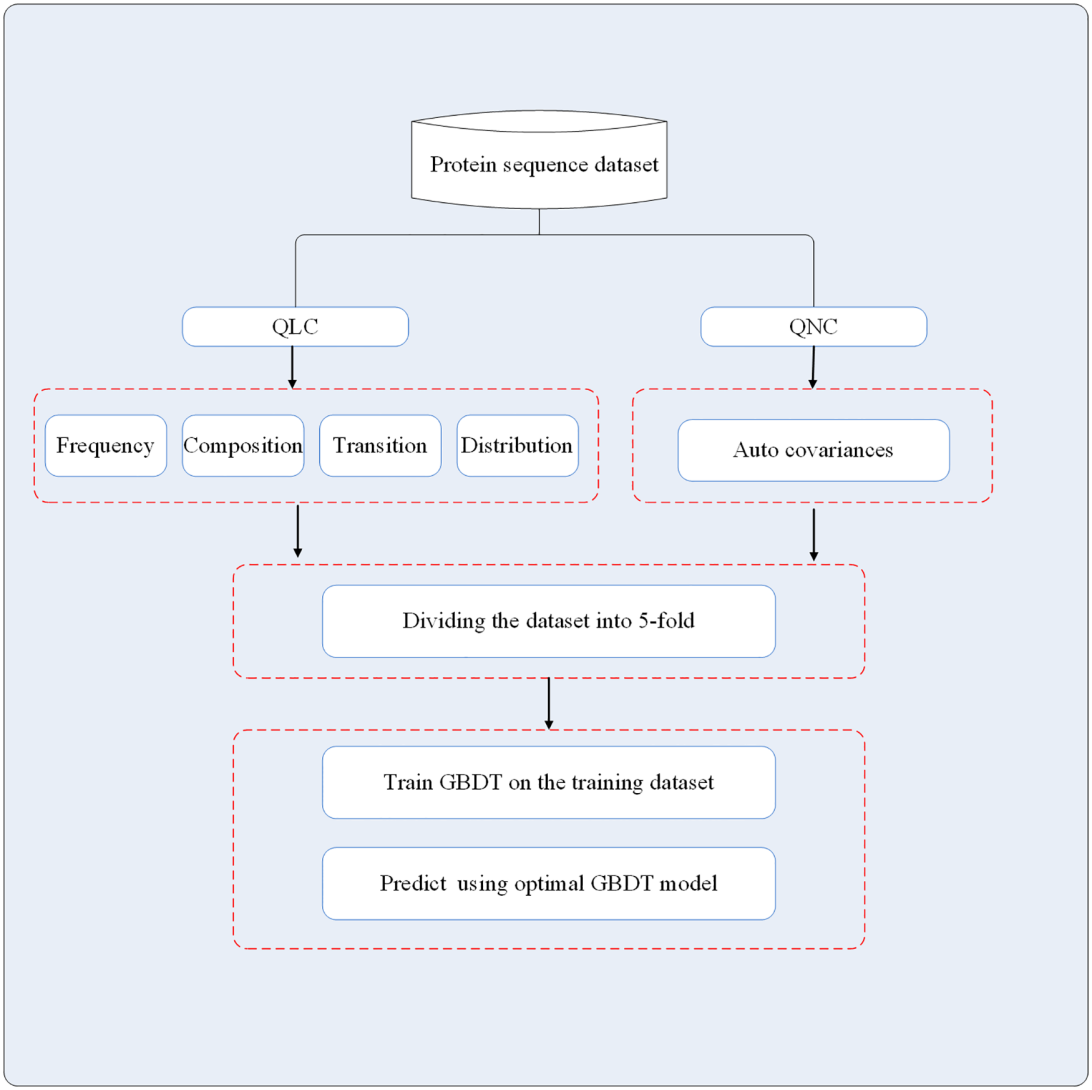
The architecture of the proposed method.

This section describes the proposed approach for predicting protein interactions from primary sequences alone. It consists mainly of three steps (see Fig 6): (1) Encode a protein sequence by qualitative and quantitative characteristics of amino acids in the sequence. (2) Extract features by five protein sequence descriptors. (3) feed feature vectors into the gradient boosting decision tree classifier for predicting PPIs.

### Encoding of protein sequences

The proposed encoding model of protein sequences is mainly based on the assumption that whether two proteins interact can be greatly influenced by their physicochemical characteristics such as residues’ hydrophobicity and polarizability. The descriptions of these properties could be qualitative or quantitative. For the first case, seven properties including hydrophobicity, normalized van der Waals volume, polarity, polarizability, charge, secondary structure, and solvent accessibility are used and each property is divided three groups, see Table 11. For the second case, six properties including hydrophobicity (H), volumes of side chains of amino acids (VSC), polarity (P1), polarizability (P2), solvent-accessible surface area (SASA) and net charge index of side chains (NCISC) are used, see Table 12.

**Table 11:** Seven physicochemical properties for 20 amino acid types.

Amino acid	Group1	Group2	Group3
Hydrophobicity	Polar	Neutral	Hydrophobicity
	R,K,E,D,Q,N	G,A,S,T,P,H,Y	C,L,V,I,M,F,W
Normalized van der Waals volume	0-2.78	2.95-4.0	4.03-8.08
	G,A,S,T,P,D	N,V,E,C,Q,I,L	M,H,K,F,R,Y,W
Polarity	4.9-6.2	8.0-9.2	10.4-13.0
	L,I,F,W,C,M,V,Y	P,A,T,G,S	H,Q,R,K,N,E,D
Polarizability	0-1.08	0.128-0.186	0.219-0.409
	G,A,S,D,T	C,P,N,V,E,Q,I,L	K,M,H,F,R,Y,W
Charge	Positive	Neutral	Negative
	K,R	A,N,C,Q,G,H,I,L,M,F,P,S,T,W,Y,V	D,E
Secondary structure	Helix	Strand	Coil
	E,A,L,M,Q,K,R,H	V,I,T,C,W,F,T	G,N,P,S,D
Solvent-accessible	Buried	Exposed	Intermediate
	A,L,F,C,G,I,V,W	R,K,Q,E,N,D	M,S,P,T,H,Y

**Table 12:** Six physicochemical properties for 20 amino acid types.

Amino acid	H	VSC	P1	P2	SASA	NCIS
A	0.62	27.5	8.1	0.046	1.181	0.007187
C	0.29	44.6	5.5	0.128	1.461	-0.03661
D	-0.9	40	13	0.105	1.587	-0.02382
E	-0.74	62	12.3	0.151	1.862	-0.006802
F	1.19	115.5	5.2	0.29	2.228	0.037552
G	0.48	0	9	0	0.881	0.179052
H	-0.4	79	10.4	0.23	2.025	-0.01069
I	1.38	93.5	5.2	0.186	1.81	0.021631
K	-1.5	100	11.3	0.219	2.258	0.017708
L	1.06	93.5	4.9	0.186	1.931	0.051672
M	0.64	94.1	5.7	0.221	2.034	0.002683
N	-0.78	58.7	11.6	0.134	1.655	0.005392
P	0.12	41.9	8	0.131	1.468	0.239531
Q	-0.85	80.7	10.5	0.18	1.932	0.049211
R	-2.53	105	10.5	0.291	2.56	0.043587
S	-0.18	29.3	9.2	0.062	1.298	0.004627
T	-0.05	51.3	8.6	0.108	1.525	0.003352
V	1.08	71.5	5.9	0.14	1.645	0.057004
W	0.81	145.5	5.4	0.409	2.663	0.037977
Y	0.26	117.3	6.2	0.298	2.368	0.0323599

**Definition 1** *A protein sequence S = s*_1_*s*_2_, ⋯, *s_n_ is encoded by a property P* = {*p*_1_, *p*_2_, *p_k_*} *if each s*_*i*_ ∈ *S is replaced by the value p_j_* ∈ *P of its corresponding property*.

Finally, for a given protein sequence, there are totally 13 kinds of encodings.

### Extraction of feature vectors

After protein sequences are encoded, feature extraction aiming at mining useful information from these encodings and represent them as fixed-length feature vectors is a crucial step for predicting protein interactions.

In this study, Five kinds of protein descriptors, amino acid frequency, composition, transition, distribution and auto covariance, are extracted to form the feature vector of a protein sequence.

The frequency of a particular amino acid in a protein sequence can be directly calculated from itself. There are 20 dimensions for this descriptor.

The composition (C), transition (T) and distribution (D), were employed to describe the global composition of each of qualitative properties.

*C* is the number of amino acids of a particular property divided by the total number of amino acids in a protein sequence. *T*characterizes the percent frequency with which amino acids of a particular property is followed by amino acids of a different property. *D* measures the chain length within which the first, 25%, 50%, 75%, and 100% of the amino acids of a particular property are located, respectively.

For each qualitative property of a protein sequence, C, T and D produce 3, 3 and 15 dimensions of features respectively. There are 7 * (3 + 3 + 15) = 147 dimensions of features for seven qualitative properties.

The features from the extraction of frequency, composition, transformation and distribution are called the qualitative characteristic feature (QLC feature) of the protein sequence in this study.

Auto covariance (AC) [9] describes the statistical significant to formalize the information of amino acids within a specific length. It accounts for the interactions between amino acids within a certain number of amino acids apart in the sequence.

For each of the six quantitative properties of amino acids in the sequence, the values of its corresponding encodings are normalized to zero mean and unit standard deviation according to the Eq 6:

$$\begin{array}{c} P_{i,j}^{{}^{\prime }}=\frac{P_{i,j}-P_{j}}{S_{j}} \end{array}$$ (6)

where *P*_*i*,*j*_ is the value of *j*-th property for *i*-th amino acid, *P*_*j*_ is the mean of *j*-th property over 20 amino acids, *S*_*j*_ is the corresponding standard deviation and *j* is the index of six quantitative properties.

Then, the AC value for *j* − *th* property of the sequence can be calculated according to the Eq 7.

$$\begin{array}{c} {\text{AC}}_{\text{lag},j}=\frac{1}{n-lag}\sum_{i=1}^{n-lag}\left(P_{i,j}-\frac{1}{n}\sum_{i=1}^{n}P_{i,j}\right)\times \left(P_{(i+lag),j}-\frac{1}{n}\sum_{i=1}^{n}P_{i,j}\right) \end{array}$$ (7)

where *n* is the length of sequence, *lag* is the length between the *i* − *th* and (*i* + *lag*) − *th* residues of the sequence. The *lag* ranges from 1 to *max* ∈ [1..*n* − 1]. Ding [24] showed that less than 30 of the *max* will lose some of the useful features while the larger may induce noises. Thus, *max* is set 30 in this study.

The number of AC values for each quantitative property is 30. Finally the AC descriptor produces 180 dimensions of features. We call this kind of features as quantitative characteristic features (QNC features).

The QLC and QNC features are directly combined to represent a protein sequence. For a pair of proteins, the feature space consists of 694 dimensions.

### Gradient boosting decision tree

As a machine learning technique for regression and classification problems, Gradient Boosting (GB) produces a prediction model in the form of an ensemble of weak prediction models, typically decision trees. Unlike common ensemble techniques such as Adaboost and random forests, the learning procedure in GB consecutively fits new models to provide a more accurate estimate of the response variables. The principle idea behind this algorithm is to build the new base learners to be maximally correlated with the negative gradient of the loss function, associated with the whole ensemble.

Supposed that there are *N* training examples: {(*x*_1_, *y*_1_), ⋯, (*x*_*N*_, *y*_*N*_)}, where *x*_*i*_ ∈ *X* ⊂ *R*^*n*^, *y*_*i*_ ∈ *Y* ⊂ *R*. The gradient boosting decision tree(GBDT) model estimates the function of future variable *x* by the linear combinition of the individual decision trees, see Eq 8.

$$\begin{array}{c} f_{M}\left(x\right)=\sum_{m=1}^{M}T\left(x;\Theta_{m}\right) \end{array}$$ (8)

Where *T*(*x*; Θ_*m*_) is the *i*-th decision tree, Θ_*m*_ is its parameter, and *M* is the number of decision trees.

The GBDT algorithm calculates the final estimation in a forward stage-wise fashion. Supposed the initial model of *x* be *f*_0_(*x*), the model in *m* step can be obtained by the Eq 9.

$$\begin{array}{c} f_{m}\left(x\right)=f_{m-1}\left(x\right)+T\left(x;\Theta_{m}\right) \end{array}$$ (9)

Where *f*_*m*−1_(*x*) is the model in *m* − 1 step. The parameter Θ_*m*_ is learned by the principle of empirical risk minimization in Eq 10.

$$\begin{array}{c} {\hat{\Theta }}_{m}=\arg\;\min_{\Theta_{m}}\sum_{i=1}^{N}L\left(y_{i},f_{m-1}\left(x\right)+T\left(x;\Theta_{m}\right)\right) \end{array}$$ (10)

Where *L* is the loss function.

Because of the assumption of linear additivity of the base function, our purpose becomes to estimate the Θ_*m*_ for best fitting the residual *L*(*y* − *f*_*m*−1_(*x*)). To this end, the negative gradient of lost function at *f*_*m*−1_ is used to estimate the residual approximately.

$$\begin{array}{c} R_{mi}=-{\left[\frac{\partial L\left(y,f\left(x_{i}\right)\right)}{\partial f\left(x_{i}\right)}\right]}_{f\left(x\right)=f_{m-1}\left(x\right)} \end{array}$$ (11)

Where *i* is the index of *i*-th example. Finally, we train a decision tree model by all the *R*_*mi*_, *i* ∈ [1..*N*] for estimating the parameter Θ_*m*_.

The parameter of a decision tree model is used to partition the space of input variables into homogeneous rectangle areas by a tree-based rule system. Each tree split corresponds to an if-then rule over some input variables. This structure of a decision tree naturally models the interactions between predictor variables. If the parameter maps the input space *X* into *J* disjoint regions *R*_1_, ⋯, *R*_*J*_, and the output is *c*_*j*_ for each region *R*_*j*_, then the tree *T* can be written as Eq 12.

$$\begin{array}{c} T\left(x;\Theta \right)=\sum_{j=1}^{J}c_{j}I\left(x_{j}\in R_{j}\right) \end{array}$$ (12)

To summarize, we can formulate the complete form of the GBDT algorithm, as in algorithm 1.

**Algorithm 1:** Gradient Boosting Decision Tree Algorithm

 **Input:** Training set *T* = {(*x*_1_, *y*_1_), ⋯, (*x*_*N*_, *y*_*N*_)} and loss function *L*(*y*, *f*(*x*)

 **Output:** The decision tree function *f*

1 Initialize $f_{0}\left(x\right)=\arg\;\min_{c}\sum_{i=1}^{N}L\left(y_{i},c\right)$

2 **for** *m* = 1, 2, ⋯, *M* **do**

3  **for** *i* = 1, 2, ⋯, *N* **do**

4   $R_{mi}=-{\left[\frac{\partial L\left(y,f\left(x_{i}\right)\right)}{\partial f\left(x_{i}\right)}\right]}_{f\left(x\right)=f_{m-1}\left(x\right)}$

5  **end**

6  Build a decision tree *T*_*m*_(*x*; Θ_*m*_) based on *R*_*mi*_, Θ_*m*_ = {*R*_*mj*_|*j* = [1..*J*]}

7  **for** *j* = 1, 2, ⋯, *J* **do**

8   $c_{mj}=\arg\;\min_{c}\sum_{x_{i}\in R_{mj}}^{N}L\left(y_{i},f_{m-1}\left(x\right)+c\right)$

9  **end**

10  Update $f_{m}\left(x\right)=f_{m-1}\left(x\right)+\sum_{j=1}^{J}c_{mj}I\left(x\in R_{mj}\right)$

11 **end**

12 $f\left(x\right)=\sum_{m=1}^{M}\sum_{j=1}^{J}c_{mj}I\left(x\in R_{mj}\right)$

13 **return** *f*(*x*)

## Conclusion

In this paper, we develop a efficient model for predicting PPIs by combining GBDT classifier with multi-scale encoding of protein sequences by the quantitative and quantitative characteristics of amino acids. The multi-scale encoding scheme is able to capture not only the compositional and positional information but also their statistical significance of amino acids in the sequence. The highly customizable GBDT classifier makes the prediction more flexible and robust. Experimental results shows that the proposed method performed significantly well in both balanced and unbalanced PPI datasets, and GBDT classier wins other classifiers. Comparative experiments demonstrate that the proposed approach outperforms all other previous methods on a couple of PPI datasets.
